# Variability of carotenoid synthesis and degradation genes in Russian durum wheat cultivars

**DOI:** 10.18699/vjgb-25-40

**Published:** 2025-06

**Authors:** A.A. Trifonova, K.V. Boris, L.V. Dedova, P.N. Malchikov, A.M. Kudryavtsev

**Affiliations:** Vavilov Institute of General Genetics, Russian Academy of Sciences, Moscow, Russia; Vavilov Institute of General Genetics, Russian Academy of Sciences, Moscow, Russia; Vavilov Institute of General Genetics, Russian Academy of Sciences, Moscow, Russia; Samara Scientific Research Agriculture Institute named after N.M. Tulajkov – Branch of Samara Federal Research Scientific Center of the Russian Academy of Sciences, Bezenchuk, Samara region, Russia; Vavilov Institute of General Genetics, Russian Academy of Sciences, Moscow, Russia

**Keywords:** yellow pigment, yellow index, molecular markers, phytoene synthase, lipoxygenase, genetic diversity, желтые пигменты, индекс желтизны, молекулярные маркеры, фитоенсинтаза, липоксигеназа, генетическое разнообразие

## Abstract

Yellow index is an important quality parameter of durum wheat cultivars, associated with carotenoid pigment content in grain and the level of carotenoid degradation during processing, and determining the yellow color of products made from durum wheat. Molecular markers of genes that influence carotenoid content can be used for fast identification of valuable genotypes and development of new high-quality durum wheat cultivars. The aim of the study was to investigate the domestic durum wheat gene pool using molecular markers of the yellow pigment synthesis (Psy-A1) and degradation (Lpx-B1) genes. Using two markers of the phytoene synthase Psy- A1 gene (PSY1-A1_STS and YP7A-2) and three markers of the lipoxygenase Lpx-B1 locus (Lpx-B1.1a/1b, Lpx- B1.1c and Lpx- B1.2/1.3), 54 durum wheat cultivars were studied for the first time. For 38 cultivars, yellow pigment content in grain was also assessed. The detected allelic variation of the phytoene synthase Psy-A1 and lipoxygenase Lpx-B1 genes was rather low. The most common Psy-A1 alleles among the studied cultivars were Psy-A1l for the PSY1- A1_STS marker and Psy-A1d for the YP7A-2 marker, identified in 51 cultivars and associated with high carotenoid content. According to the markers of the Lpx-B1 locus, haplotype II, associated with medium lipoxygenase activity, identified in 43 cultivars, was predominant. Haplotype III, associated with low enzyme activity, was identified in only three winter durum wheat cultivars (Donchanka, Gelios and Leucurum 21). Despite the predominance of allelic variants associated with increased carotenoid content and moderate lipoxygenase activity, the studied cultivars had different levels of yellow pigment content in grain, from low to high.

## Abstract (ru)

Индекс желтизны – важный параметр качества сортов твердой пшеницы, связанный с содержанием каротиноидов в зерне и уровнем их деградации в процессе его переработки и определяющий желтый цвет продуктов, получаемых из твердой пшеницы. Применение молекулярно-генетических маркеров генов, влияющих на содержание каротиноидов, позволяет быстро идентифицировать ценные для селекции генотипы для ускоренного создания новых высококачественных отечественных сортов твердой пшеницы. Целью работы стало изучение отечественного генофонда твердой пшеницы с помощью молекулярных маркеров генов синтеза (Psy-A1) и деградации (Lpx-B1) желтых пигментов в зерне. С использованием двух маркеров гена фитоенсинтазы Psy-A1 (PSY1-A1_STS и YP7A-2) и трех маркеров локуса липоксигеназы Lpx-B1 (Lpx-B1.1a/1b, Lpx-B1.1с и Lpx-B1.2/1.3) впервые были исследованы 54 сорта твердой пшеницы, 38 из которых охарактеризованы по уровню содержания желтых пигментов в зерне. Аллельное разнообразие изученных отечественных сортов твердой пшеницы по генам фитоенсинтазы Psy-A1 и липоксигеназы Lpx-B1 оказалось достаточно низким. Наиболее распространенными в выборке были аллельные варианты Psy-A1l по маркеру PSY1-A1_STS и Psy-A1d по маркеру YP7A-2, выявленные у 51 образца и ассоциированные с высокими значениями индекса желтизны. По маркерам локуса Lpx-B1 в выборке преобладал гаплотип II, связанный со средней активностью липоксигеназы, который был идентифицирован у 43 образцов. Гаплотип III, ассоциированный с низкой активностью фермента, выявлен только у трех озимых сортов (Дончанка, Гелиос и Леукурум 21). Несмотря на преобладание аллельных вариантов, связанных с повышенным содержанием каротиноидов и средней активностью липоксигеназы, исследуемые образцы имели различный уровень содержания желтых пигментов – от низкого до высокого

## Introduction

Durum wheat (Triticum durum Desf.) is an important cereal crop. Hardness, amber-yellow color and high content of protein and gluten in durum wheat grain allow making high-quality pasta, as well as semolina, bulgur and couscous (Shevchenko et al., 2018). In Russia, about 650–700 thousand tons of durum wheat are produced annually. Currently, the domestic market’s demand for this crop is growing and is estimated at 1.5 million tons (Natoli et al., 2021). At the same time, our country has the capacity to meet the growing need for durum wheat, as well as exports. There is enough arable land, and the conditions of the Volga, Siberia and Urals steppe regions allow to produce a sufficient amount of highquality durum wheat grain (Shevchenko et al., 2018; Natoli et al., 2021). Currently, the State Register of Varieties and Hybrids of Agricultural Plants Admitted for Usage (National list) (2024) includes 71 spring and 37 winter durum wheat cultivars, adapted to various growing regions. Developing new domestic cultivars with high quality parameters for pasta production that follow international standards will help to satisfy the growing demand of processing companies.

Yellow index is one of the main quality parameters of durum wheat grain affecting the yellow color of pasta, which is important to consumers (Colasuonno et al., 2019; Requena- Ramirez et al., 2022). Yellow index largely depends on the genotype, so developing domestic cultivars with high yellow index is justified and relevant (Malchikov, Myasnikova, 2020).

Yellow index is a complex trait that is associated with the content of yellow pigments, mainly carotenoids, in grain and the level of their degradation during processing (Colasuonno et al., 2019; Parada et al., 2020). Carotenoids not only provide the yellow color of the grain and its end products, but are also important for human nutrition, as precursors of vitamin A (Ficco et al., 2014). There is a significant positive correlation between yellow index and yellow pigment content in grain, and these indicators are often used to characterize the color of durum wheat grain and end products (Digesu et al., 2009; Blanco et al., 2011; Campos et al., 2016).

In durum wheat breeding in Russia, there has been a significant increase in the yellow index, especially in recently released cultivars (Vasil’chuk, 2001; Malchikov, Myasnikova, 2020). However, at present, breeding centers working on increasing carotenoid concentration in grain, semolina and end products mainly use traditional breeding methods. To accelerate the breeding process, it is necessary to use modern molecular genetic methods, e. g., for the identification of alleles associated with high yellow index.

Phytoene synthase (PSY, EC 2.5.1.32) is the major enzyme of carotenoid accumulation in the endosperm, which catalyzes the first stage of carotenoid biosynthesis (Gallagher et al., 2004). Of the three known PSY isoforms, PSY-1, which is active in maturing grain as well as in young leaves, plays the most important role. As previously shown, the Psy-A1 and Psy-B1 genes encoding PSY-1 are located on chromosomes 7A and 7B respectively and are linked to the major QTLs associated with yellow pigment content in durum wheat. Of the two genes, Psy-A1 has a greater influence on carotenoid content, explaining up to 50 % of phenotypic variability (Colasuonno et al., 2019). Several allelic variants of the Psy-A1 gene associated with insertions/deletions in the third and fourth introns and related to different carotenoid content in grain have been identified in common and durum wheat (He et al., 2008, 2009a; Singh et al., 2009).

Various markers (YP7A, YP7A-2, PSY1-A1_STS, Psy- A1SSR) have been developed to identify alleles of the Psy-A1 gene (He et al., 2008, 2009a, b; Singh et al., 2009; Patil et al., 2018). These markers were previously used to study the allelic diversity of the phytoene synthase gene in landraces and modern foreign wheat cultivars (Singh et al., 2009; Campos et al., 2016; Parada et al., 2020), as well as in durum wheat breeding lines (Campos et al., 2016; Patil et al., 2018). The association of the identified allelic variants with different levels of yellow index was confirmed, and effectiveness of these markers for breeding was shown (Campos et al., 2016).

One of the main enzymes leading to the degradation of carotenoids during durum wheat grain processing and the bleaching of the end products is lipoxygenase (LOX, EC 1.13.11.12), which catalyzes the oxidation of polyunsaturated fatty acids (Verlotta et al., 2010; Colasuonno et al., 2019). Of the loci encoding various lipoxygenase isoforms in durum wheat (Lpx-1, Lpx-2, Lpx-3), the Lpx-B1 locus plays the major role at the final stages of grain maturation, accounting for 36 to 54 % of the enzyme activity variation (Carrera et al., 2007; Verlotta et al., 2010; Parada et al., 2020). The Lpx-B1 locus is located on the short arm of chromosome 4B and includes three related genes: Lpx-B1.1, Lpx-B1.2 and Lpx-B1.3 (Verlotta et al., 2010). The differences between these genes and their allelic variants are due to the presence of DNA transposon of the MITE (Miniature Inverted-Repeat Transposable Element) group (Hessler et al., 2002; Carrera et al., 2007), the transposition of which led to a large deletion in the sequence of the Lpx-B1.1 gene and a significant decrease in lipoxygenase activity (Carrera et al., 2007; Verlotta et al., 2010). Several molecular markers have been developed to identify the genes of the Lpx-B1 locus and their allelic variants (Verlotta et al., 2010; Parada et al., 2020). In previous studies of foreign durum wheat cultivars using these markers, several different combinations between the alleles and genes of the Lpx-B1 locus (haplotypes) associated with different levels of lipoxygenase activity were reported (Verlotta et al., 2010; Parada et al., 2020).

The use of the mentioned markers of phytoene synthase and lipoxygenase genes to study domestic durum wheat material will allow to characterize its allelic diversity for the first time and to assess its potential for breeding. The use of appropriate markers for the selection of breeding material and the involvement of genotypes with target alleles into the breeding process will significantly accelerate the development of durum wheat cultivars with high-quality grain.

The aim of the work was to study domestic durum wheat cultivars differing in the level of yellow pigment content using molecular markers of the Psy-A1 gene and the Lpx-B1 locus and to compare the results with data on the variability of the foreign durum wheat gene pool.

## Materials and methods

Plant material. For the study, 54 spring and winter durum wheat cultivars from the collections of the Samara Federal Research Scientific Center, Russian Academy of Sciences and Vavilov Institute of General Genetics of the Russian Academy of Sciences were selected (Table 1). Of the selected cultivars, 44 (two foreign and 42 domestic cultivars from various breeding centers) are included in the State Register of Varieties and Hybrids of Agricultural Plants Admitted for Usage (National list) (2024). Cultivars Langdon and Giusto were used as references

**Table 1.**
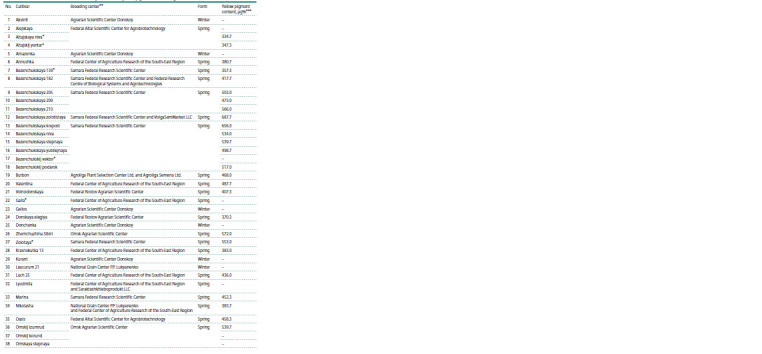
Durum wheat cultivars used in the study and data on yellow pigment content in grain

**Table 1end.**
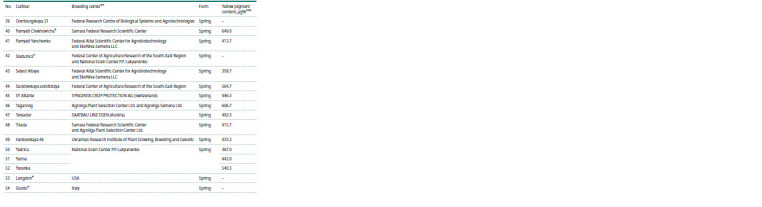
Table 1end.

DNA was isolated from five-day-old seedlings according to the standard CTAB protocol (Doyle J.J., Doyle J.L., 1990) with minor modifications. For each cultivar, two DNA samples from individual plants were obtained, and further analysis was carried out with two repetitions

Phytoene synthase (Psy-А1) and lipoxygenase (Lpx- B1) gene markers. Genotyping of the studied cultivars was carried out using SCAR markers of the Psy-A1 and Lpx-B1 genes. The primer sequences and annealing temperatures are presented in Table 2.

**Table 2.**
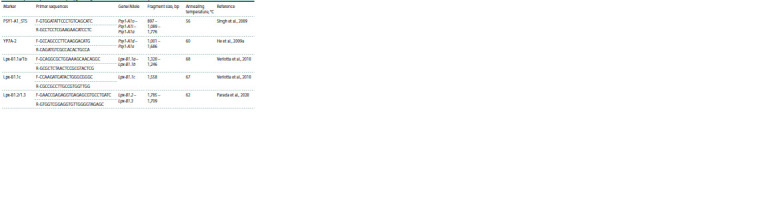
Phytoene synthase and lipoxygenase gene markers used in this study

PCR reactions were performed in a GeneAmp 9700 (Applied Biosystems, USA) thermal cycler. PCR reaction mixture 15 μl in volume contained 20 ng of genomic DNA, 0.3 μM of each primer (Syntol, Russia), 0.16 mM dNTPs, 1.6 mM MgCl2, 1 u Taq polymerase and 1x standard PCR buffer (Dialat LTD., Russia). To determine PCR fragment sizes, GeneRuler 100 bp DNA ladder (Thermo Fisher Scientific, USA) was used. After amplification, PCR products were separated in 1.5 % agarose gels, stained with ethidium bromide, analyzed on a UV-light box and photographed.

Yellow pigment content. For 38 cultivars from the Samara Federal Research Scientific Center of the Russian Academy of Sciences, total yellow pigment content in grain was assessed (Table 1). To assess the yellow pigment content, cultivars were grown for three years, from 2021 to 2023, in the Samara Scientific Research Agriculture Institute field. The evaluation of yellow pigment content was made by extraction of total pigment in water-saturated n-butanol followed by photocolorimetric quantification of the absorbance of extract at 440–450 nm using a KFK-3 M spectrophotometer. For each sample, 7.0 g of semolina were taken, placed in a 20 × 220 mm tube with a stopper, which was then filled with 35 ml of water-saturated n-butanol, shaken vigorously for one minute and left for extraction in a darkened room for 18 hours at room temperature. Then the solution was filtered through a pleated filter into clean tubes. The yellow pigment content was evaluated using a spectrophotometer in a cuvette with a working distance of 10 mm. The pigment content in parts per million parts of semolina (ppm) was calculated by multiplying the obtained value by a coefficient of 16.632. For convenience, the obtained value was converted into microgram percent by multiplying it by 100 (100 μg% = 1 ppm) (Methods for Assessing…, 1971). Measurements were taken for each of the two plants of one cultivar, and then the average value was determined. The yellow pigment content was considered high if it was more than 500 μg%, intermediate – 401–500 μg%, and low – 200–400 μg%.

The influence of environmental conditions in different years was assessed based on the average value of pigment content in grain in the experiment. According to this principle, the years were arranged in the following order: 2022 with the maximum (546.9 μg%), 2021 with the intermediate (476.2 μg%), 2023 with the minimum pigment content (402.1 μg%). The results were analyzed by the two-ways analysis of variance (ANOVA) using MS Excel. The parameters of general, specific adaptability (GACi, SACi) and stability (Sgi) of the trait were calculated according to the method of A.V. Kilchevsky, L.V. Khotyleva (1997). The regression coefficient (bi) that measures the response of the cultivar to varying environments was determined following S.A. Eberhart, W.A. Russell (1966) as presented by A.V. Kilchevsky, L.V. Khotyleva (1997).

## Results

In the present study, 54 durum wheat cultivars were analyzed using two markers of the Psy-A1 phytoene synthase gene: PSY1-A1_STS and YP7A-2, and three markers of the Lpx-B1 lipoxygenase locus: Lpx-B1.1a/1b, Lpx-B1.1c and Lpx-B1.2/1.3 (Table 2). Clear and reproducible results were obtained for all samples, coinciding for the two studied samples of each cultivar.

The PSY1-A1_STS marker identifies alleles Psy-A1a (1,776 bp), Psy-A1l (1,089 bp) and Psy-A1o (897 bp). Cultivar Langdon, for which the presence of the Psy-A1l allele was previously shown (Singh et al., 2009), was used as a reference.

**Fig. 1.**
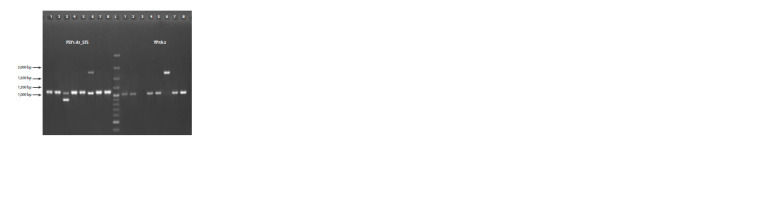
Results of the Psy-A1 alleles identification with markers PSY1-A1_STS and YP7A-2 in durum wheat cultivars: 1 – Aksinit; 2 – Alejskaya; 3 – Donchanka; 4 – Zhemchuzhina Sibiri; 5 – Zolotaya; 6 – Kurant; 7 – Leucurum 21; 8 – Langdon; L – marker GeneRuler 100 bp Plus.

Using the PSY1-A1_STS marker, the Psy-A1l allele was detected in 51 studied cultivars, including Langdon. The Psy- A1o allele was identified in two cultivars Krasnokutka 13 and Donchanka, and the Psy-A1a allele in cultivar Kurant (Fig. 1, Table 3). In cultivars Krasnokutka 13, Donchanka and Kurant, an additional ~1,100 bp fragment was amplified with the PSY1-A1_STS marker, which was not taken into account in further analysis (Fig. 1). It was previously shown that the presence of an additional ~1,100 bp fragment together with the Psy-A1o or Psy-A1a alleles occurs due to cross-amplification of the Psy-B1n allele of the Psy-B1 locus (Singh et al., 2009; Campos et al., 2016).

**Table 3.**
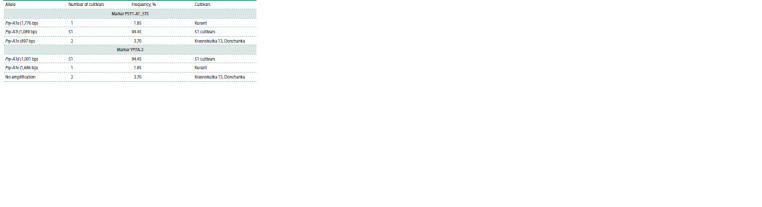
Alleles of the Psy-A1 gene identified in the studied durum wheat cultivars

The YP7A-2 marker allows detection of the Psy-A1d (1,001 bp) and Psy-A1e (1,686 bp) alleles. Cultivar Langdon, for which the presence of the Psy-A1d allele was previously shown (He et al., 2009b), was used as a reference.

With the YP7A-2 marker, the Psy-A1d allele was identified in 51 cultivars, including Langdon, the Psy-A1e allele was identified in Kurant, and the absence of amplification products was detected in Krasnokutka 13 and Donchanka (Fig. 1, Table 3).

Analysis of the Lpx-B1.1, Lpx-B1.2 and Lpx-B1.3 lipoxygenase genes variability in 54 durum wheat cultivars was also performed. The allelic state of the Lpx-B1.1 gene was analyzed using two markers: Lpx-B1.1a/1b was used to distinguish between the Lpx-B1.1a (1,320 bp) and Lpx-B1.1b (1,246 bp) alleles, and Lpx-B1.1c, to identify the Lpx-B1.1c allele (1,558 bp) (Verlotta et al., 2010). To identify the Lpx-B1.2 (1,785 bp) and Lpx-B1.3 (1,709 bp) genes, the Lpx-B1.2/1.3 marker (Parada et al., 2020) was used (Table 2). Cultivar Giusto, for which the presence of the Lpx-B1.2 gene and the Lpx-B1.1c allele was previously shown (Verlotta et al., 2010), was used as a reference.

**Fig. 2.**
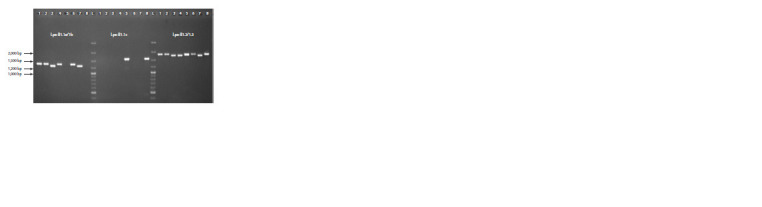
Results of the Lpx-B1 locus genes and alleles identification with markers Lpx-B1.1a/1b, Lpx-B1.1c and Lpx-B1.2/1.3 in durum wheat cultivars: 1 – Bezenchukskaya 209; 2 – Bezenchukskaya 210; 3 – Bezenchukskaya zolotistaya; 4 – Alejskaya; 5 – Donchanka; 6 – Zhemchuzhina Sibiri; 7 – Pamyati Chekhovicha; 8 – Giusto; L – marker GeneRuler 100 bp Plus.

Allele Lpx-B1.1a was identified in 45 cultivars, Lpx-B1.1b, in five cultivars (Bezenchukskaya zolotistaya, Bezenchukskij vector, Bezenchukskij podarok, Pamyati Chekhovicha and Saratovskaya zolotistaya), and Lpx-B1.1c, in four cultivars (Gelios, Donchanka, Leucurum 21 and Giusto) (Fig. 2, Table 4).

**Table 4.**
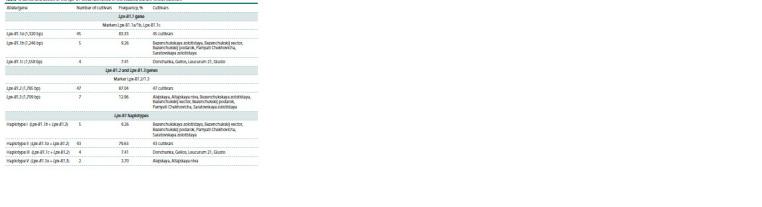
Genes and alleles of the Lpx-B1 locus identified in the studied durum wheat cultivars

Using the Lpx-B1.2/1.3 marker, the Lpx-B1.2 gene was detected in 47 cultivars, and the Lpx-B1.3 gene, in seven cultivars (Alejskaya, Altajskaya niva, Bezenchukskaya zolotistaya, Bezenchukskij vector, Bezenchukskij podarok, Pamyati Chekhovicha, and Saratovskaya zolotistaya) in the studied collection (Fig. 2, Table 4).

Using markers of the Lpx-B1 locus to analyze foreign durum wheat cultivars and breeding lines, five haplotypes with different combinations of the Lpx-B1.1 gene alleles and one of In the studied set, 14 cultivars had high (more than 500 μg%), 15 cultivars had medium (400–500 μg%), and nine cultivars had low yellow pigment content (200–400 μg%) (Table 1).

The relative influence of genotype, environmental conditions (in this experiment, conditions of the year) and their interaction on the accumulation of yellow pigment in grain was determined using 38 spring durum wheat genotypes in a 3-year (2021–2023) experiment at the Samara Federal Research Scientific Center of the Russian Academy of Sciences. As a result, significant effects of all factors were established using the two-way analysis of variance. The contributions of genotype, environment and their interaction to the total variance were 65.3, 28.0 and 6.3 %, respectively (Supplementary Materials, Table S1)1


Supplementary Materials are available in the online version of the paper: https://vavilov.elpub.ru/jour/manager/files/Suppl_Trifonova_Engl_29_3.pdf


On average, for the groups of cultivars with medium and high values, the parameters of general and specific adaptability (GACi, SACi), responsiveness to the environment (by the regression coefficient – bi) of the trait “yellow pigment content in grain” significantly exceeded similar parameters for the group with a low value of the trait. Judging by the regression coefficient, the most effective assessment of the phenotype can be given in favorable environmental conditions. At the same time, no significant differences were observed between the groups for the relative stability parameter (Sgi) (Table S2).

The rank correlation coefficients between the cultivars’ arrangement in the variability rows by the content of yellow pigment by years and between the ranks of cultivars by average values for three years and for each year varied within 0.83–0.96, which is significant at the 1.0 % level. These results suggest that the studied set of spring durum wheat genotypes differs significantly in the accumulation of yellow pigment in grain, the differences between cultivars are stable under different environmental conditions, and this is the result of the functioning of the corresponding genetic systems.

## Discussion

The analysis of 54 durum wheat cultivars using markers of the Psy-A1 phytoene synthase gene and the Lpx-B1 lipoxygenase locus allowed evaluating their variability in the studied collection.

The markers were used for the first time to analyze domestic durum wheat cultivars. Fragments of the expected size were obtained with all markers and allelic variants previously described when analyzing foreign material were identified. The results were clear, reproducible, and coincided for the two studied samples of each cultivar, which indicates the effectiveness of using these markers to analyze domestic durum wheat cultivars.

Analysis of the Psy-A1 phytoene synthase gene polymorphism

To analyze the Psy-A1 gene, encoding a key enzyme of carotenoid synthesis, two SCAR markers, PSY1-A1_STS and YP7A-2, were used, in order to identify differences between alleles having indels in the third and fourth introns associated with the level of yellow pigment content (He et al., 2009a; Singh et al., 2009).

The study of the collection using these markers showed an extremely low level of its diversity. The Psy-A1l allele (PSY1-A1_STS marker) prevailed, as well as the Psy-A1d allele (YP7A-2 marker). These alleles were noted in 51 cultivars studied; their frequency was 94.45 %. Only three cultivars had other Psy-A1 alleles. In cultivars Krasnokutka 13 and Donchanka, the Psy-A1o allele was identified with the PSY1-A1_STS marker, and no amplification with the YP7A- 2 marker was noted, and in the cultivar Kurant, the Psy-A1a allele was detected with the PSY1-A1_STS marker, and the Psy-A1e, with the YP7A-2 marker (Table 3).

The combined use of the PSY1-A1_STS and YP7A-2 markers showed the correspondence of the detected allelic variants, which was also noted in previous studies (Campos et al., 2016; Patil et al., 2018). Thus, samples having the Psy- A1l allele identified with the PSY1-A1_STS marker had the Psy- A1d allele detected with the YP7A-2 marker, and samples having the Psy-A1a allele identified with the PSY1-A1_STS marker had the Psy-A1e allele detected with the YP7A-2 marker. When Psy-A1o was detected with the PSY1-A1_STS marker, there was no amplification with the YP7A-2 marker. Thus, both markers allow to detect the 688 bp indel in the fourth intron of the Psy-A1 gene, which can distinguish the Psy-A1l and Psy-A1d alleles from Psy-A1a and Psy-A1e. Using the PSY1-A1_STS marker, an additional Psy-A1o allele can be identified, which is not detected using the YP7A-2 marker due to a 198 bp deletion in the third intron, which results in the absence of the binding site for the forward primer of the YP7A-2 marker (Campos et al., 2016).

Previously, when studying durum wheat collections using Psy-A1 gene markers, an association of the identified alleles with the level of yellow pigment content was shown. Alleles Psy-A1d and Psy-A1e, identified using the YP7A-2 marker, were associated with high and low yellow index, respectively (He et al., 2009b). Alleles Psy-A1l and Psy-A1o, identified using the PSY1-A1_STS marker, were associated with high or intermediate, and Psy-A1a, with low content of yellow pigment (Singh et al., 2009; Campos et al., 2016).

Thus, in the studied collection, Psy-A1 alleles associated with high and intermediate yellow pigment content (Psy-A1l, Psy-A1o and Psy-A1d) predominate. Alleles associated with low yellow index (Psy-A1a and Psy-A1e) were identified only in one cultivar.

Similar results were shown in studies of the foreign durum wheat gene pool. So, in the collections of foreign cultivars released in different periods, as well as in breeding lines studied using the PSY1-A1_STS marker, the Psy-A1l allele prevailed with a 68 to 97 % frequency (Singh et al., 2009; Campos et al., 2016; Parada et al., 2020). In the study of 100 durum wheat breeding lines from the CIMMYT collection using the YP7A-2 marker, the prevalence of the Psy-A1d allele was revealed (99 % frequency). Allele Psy-A1o was quite common in Mediterranean landraces, but was rare in modern cultivars, despite its association with a high or intermediate yellow index (Campos et al., 2016). Alleles associated with low yellowness were also rare in the foreign gene pool (Singh et al., 2009; Campos et al., 2016; Parada et al., 2020).

The predominance of allelic variants associated with high yellow pigment content may be the result of a long selection process that led to the rejection of samples with alleles that negatively affect the trait

Analysis of the Lpx-B1 lipoxygenase locus polymorphism

Using three SCAR markers Lpx-B1.1a/1b, Lpx-B1.1c and Lpx-B1.2/1.3, haplotypes of the Lpx-B1 locus were determined for all cultivars studied. It was previously shown that of the five Lpx-B1 haplotypes, only haplotype III is associated with low lipoxygenase activity (Verlotta et al., 2010; Parada et al., 2020).

Four of the five previously reported haplotypes were identified in the studied cultivars, with haplotype II, associated with an intermediate level of lipoxygenase activity, being the most common and occurring with 79.63 % frequency (Table 4). Among foreign durum wheat cultivars, this haplotype was also quite common; for example, in Mediterranean landraces, the frequency of this haplotype was 54 % (Parada et al., 2020), and in cultivars of different breeding periods cultivated in Italy, 42 % (Verlotta et al., 2010).

The most valuable for breeding is haplotype III (the Lpx-B1.1c allele and the Lpx-B1.2 gene). Due to the MITE transposition, a large deletion occurred in the sequence of the Lpx-B1.1c allele, which led to the loss of gene function and a significant decrease in lipoxygenase activity (Carrera et al., 2007; Verlotta et al., 2010). Haplotype III was identified in only three studied winter cultivars: Donchanka, Gelios, and Leucurum 21, and was not found among spring cultivars. Previously, in a study of 85 predominantly Italian durum wheat genotypes released in different breeding periods (before 1971; 1971–1990; 1991–2005), this haplotype was noted in 41 cultivars, 32 of which were released after 1991 (Verlotta et al., 2010). Among Italian cultivars of an earlier breeding period, haplotype III was much less common (Verlotta et al., 2010), and the frequency of this haplotype was also low in Mediterranean landraces (Parada et al., 2020).

Haplotype I, associated with high lipoxygenase activity, was identified in four cultivars from the Samara Federal Research Scientific Center of the Russian Academy of Sciences: Bezenchukskaya zolotistaya, Bezenchukskij podarok, Bezenchukskij vector, Pamyati Chekhovicha and Saratovskaya zolotistaya from the Federal Center of Agriculture Research of the South-East Region. Among Mediterranean landraces, the frequency of this haplotype was 39 %, and in cultivars grown in Italy, this haplotype was found mainly in the material released before the 1970s, and was not found in modern cultivars (Verlotta et al., 2010; Parada et al., 2020).

Cultivars Alejskaya and Altajskaya niva from the Federal Altai Scientific Center for Agrobiotechnology had haplotype V. This haplotype is associated with high lipoxygenase activity and is quite rare in foreign cultivars (Parada et al., 2020).

In general, according to previous studies, in the foreign gene pool, the proportion of the Lpx-B1 haplotype III, valuable for breeding, increases in modern cultivars and breeding lines, which indicates targeted selection of cultivars with a low level of lipoxygenase activity. At the same time, in the domestic gene pool, the frequency of haplotype III is still quite low. The use of Lpx-B1 locus markers for the analysis of domestic breeding material will contribute to the effective selection of genotypes with haplotype III

Association between yellow pigment content and identified alleles of the Psy-A1 gene and haplotypes of the Lpx-B1 locus

The studied cultivars varied significantly in the accumulation of yellow pigment in grain (Table 1). Most of the cultivars had medium and high content of yellow pigment. At the same time, according to molecular markers, most cultivars, including those with low yellow pigment content, were found to have Psy-A1 alleles that determine high yellowness (Psy-A1d and Psy-A1l), as well as haplotype II of the Lpx-B1 locus, associated with an intermediate level of lipoxygenase activity. Such a discrepancy may be due to the fact that the yellow index is a complex, polygenic trait that depends on the interaction of various enzymes, controlling both carotenoid synthesis and degradation (Colasuonno et al., 2019). Furthermore, the haplotype of the lipoxygenase locus has a greater influence on the trait at post-harvest stages and during pasta manufacturing (flour and pasta yellow index) (Parada et al., 2020).

Also, according to the data obtained, 65.3 % of the trait variance was determined by the genotype. The significant prevalence of the genotype effect over the influence of the environment and the genotype–environment interaction confirms data on the high heritability of the yellow pigment accumulation processes in durum wheat grain with the predominance of additive effects of genes (Blanco et al., 2011; Roncallo et al., 2012; Schulthess, Schwember, 2013).

## Conclusion

Thus, using molecular markers, the allelic diversity of the Psy-A1 phytoene synthase and Lpx-B1 lipoxygenase genes in Russian durum wheat cultivars was studied for the first time, and turned out to be quite low. In the studied cultivars, allelic variants of the Psy-A1 gene associated with high yellow pigment content predominate, as in most modern foreign durum wheat cultivars. Haplotype III of the Lpx-B1 locus, valuable for breeding, associated with low lipoxygenase activity, was detected only in three winter cultivars (Donchanka, Gelios and Leucurum 21), while among foreign cultivars, especially modern ones, the proportion of this haplotype is significantly higher. The obtained results confirmed the dependence of the yellow pigment content on the genotype; however, the presence of the Psy-A1 and Lpx-B1 alleles associated with high carotenoid content did not always determine their high content in the grain of the studied cultivars, which is most likely due to the influence of other genes of yellow pigment metabolism. Nevertheless, the studied markers can be used for breeding new durum wheat cultivars with a high yellow index.

## Conflict of interest

The authors declare no conflict of interest.
